# The Dawn of a New Common

**DOI:** 10.1007/978-3-030-65355-2_1

**Published:** 2021-03-20

**Authors:** Emile Aarts, Hein Fleuren, Margriet Sitskoorn, Ton Wilthagen

**Affiliations:** 1 Tilburg University, Tilburg, The Netherlands; 2 Tilburg University, Tilburg, The Netherlands; 3 Tilburg University, Tilburg, The Netherlands; 4 Tilburg University, Tilburg, The Netherlands; 5 Department of Econometrics and Operations Research, Tilburg School of Economics and Management, Tilburg, The Netherlands; 6 Department of Cognitive Neuropsychology, Tilburg School of Social and Behavioral Sciences, Tilburg, The Netherlands; 7 Department of Public Law and Governance, Tilburg Law School, Tilburg, The Netherlands

## Abstract

On Thursday, February 27, 2020, during a live broadcast on television, Minister Bruno Bruin is handed a note saying that it has just been confirmed that a patient with the coronavirus has been identified in the Netherlands. Allegedly, it concerns a man who is placed in isolation in the Elisabeth-Tweesteden hospital in Tilburg.

This is where the story of our book starts. The hospital mentioned by the minister is hardly a kilometer away from our university, Tilburg University. Things now start to develop quickly. During several weeks, the region of Tilburg becomes the “Corona Capital” of the Netherlands in terms of the number of people infected. On March 18, Minister Bruno Bruins collapses due to exhaustion during a debate in the Government’s House of Representatives. The following day, he resigns and soon after is temporarily replaced by a politician of a party that is not part of the current political coalition. Two days earlier, the country had gone into a lockdown after a historical speech of Dutch Prime Minister Mark Rutte.

Thursday, February 27, 2020. Dutch National television is broadcasting a special information program on the COVID-19 virus that has hit several countries, also in Europe, after the initial outbreak in the Chinese city of Wuhan. So far, no patients have been registered in the Netherlands. It is 09:20 pm and the program has been running for exactly 50 min. The main guest is Minister of Health, Welfare, and Sport Bruno Bruins. After a short video about the virus, the program host Rob Trip suddenly says: “Mr. Bruins, you are being handed a note!” The minister speaks up: It has just been confirmed that a patient with the coronavirus has been identified in the Netherlands. Allegedly, it concerns a man who is placed in isolation in the Elisabeth-Tweesteden hospital in Tilburg.

This is where the story of our book starts. The hospital mentioned by the minister is hardly a kilometer away from our university, Tilburg University. Things now start to develop quickly. During several weeks, the region of Tilburg becomes the “Corona Capital” of the Netherlands in terms of the number of people infected. On March 18, Minister Bruno Bruins collapses due to exhaustion during a debate in the Government’s House of Representatives. The following day, he resigns and soon after is temporarily replaced by a politician of a party that is not part of the current political coalition. Two days earlier, the country had gone into a lockdown after a historical speech of Dutch Prime Minister Mark Rutte.

## It Turns Out to Be a Pandemic and Quite Scary

At the time of writing, July 1, 2020, the official number of persons positively tested with COVID-19 in the Netherlands amounted to 50,147; 11,871 persons infected by the virus had been admitted to a hospital; and 6105 persons had died. It started as a local breakout in the city of Wuhan in China in December last year and developed overwhelmingly rapidly into a worldwide disaster. Unlike the SARS epidemic in 2003, COVID-19 spreads around the world at an unprecedented pace, and on March 11, 2020, the World Health Organization officially declared it a pandemic. By July 15, 2020, people in some 190 countries around the world were affected, almost ten million people were infected and about half a million persons died.

The common view to fight the coronavirus crisis is to bring the so-called reproduction rate R (the average number of people who will contract a contagious disease from one person with that disease) below 1. This can be achieved with a vaccine, but we do not have one yet. We stimulate science and the pharmaceutical industry to give their utmost effort to find one. In the meantime, we have to maintain social distancing, and this has a major impact on our society. Worldwide, countries have responded to the COVID-19 pandemic in different ways, but quarantines and lockdowns have become general response measures taken by authorities at various levels: local, regional, and national. It is this type of measure that is impacting our society in all its dimensions. Many believe, for example, that this will put an end to globalization for a long time. Massive transportation of persons and goods will be reduced substantially. Education will change. National governments, and consequently the role of nationalism, might regain significance, and unemployment and poverty numbers will rise.

Paolo Giordano (2020) was one of the first to describe the effects of social isolation in his essay Nel Contagioi. He was also one of the first to share the concerns that frightened him with a broader audience through his touching report of what he experienced during his personal quarantine. He came to the conclusion that the worldwide spread of the virus shows that our society has become truly global over the past decades, with all its drawbacks. He stresses that the coronavirus crisis affects the entire world and that the only way to prevail is to come up with a collaborative approach starting at the level of our individual lives ranging up to the level of our planet as a global organism.

Clearly, COVID-19 is not the first pandemic that has hit humankind. Our memory of pandemics, however, is not well developed because we did not experience firsthand what happened in earlier times and do not learn easily from descriptions and therefore fail to profit from the lessons that could be drawn from earlier pandemics. In her riveting book, the journalist Laura Spinney (2018) explains how the Spanish Flue of 1918 has dramatically changed the world while it is one of the most widely denied global events of the past century costing more lives than both world wars together. As an explanation, she argues that we do not like the thought of people dying in a terrible way through suffocation and without reason or sensemaking.

Other than during World War I and II, there are no clear and obvious opponents. The coronavirus acts as an invisible assassinator and equalizer and anyone can become a victim. Consequently, after the crisis is over, we are all survivors. Admittedly, we all understand how it started in December 2019, but the way it will end is unclear and that is what people find hard to deal with. We resort to science to explain to us what is happening, to tell us which of the many scenarios will most likely develop and what to do and what not to do, but we feel uncertain and disoriented. Science turns out to be imperfect as virologists and epidemiologists produce conflicting theories and statements, or some of us, including political leaders, just do not like the implications of their advice and go into denial. Whom should we believe in trying to find a way out?

At the same time, states take over control and leadership. Kleinfeld (2020) analyses the worldwide difference between the approaches the various countries take to handle and fight the crisis. There are more or less authoritarian states that seem to be successful in their approach, such as China, Singapore, and South Korea. On the other hand, there are the democratic states but they also show different levels of success in their approaches. The USA, Brazil, and the UK fail as their measures are inadequate and late; Italy ran into problems very early and was overwhelmingly impacted by the virus whereas Germany and New Zeeland clearly seem to be successful. This brings us to the central observation that not only the type of government determines whether the approach is successful, but also the trust citizens put in their governments and the measures taken.

As we write down these words, the world is expecting one of the deepest social and economic recessions in modern history. Apparently, an ecological crisis has turned into a health crisis, which, in turn, has transformed into a socioeconomic crisis. And again, as the Dutch writer Geert Mak (2020) argues with an imaginary student of history in the year 2069, we did not see it coming in the Global North being used to our seemingly smooth and undisturbed way of living.

## Replacing an “Old Common” With a “New Common”

In this introduction, we hypothesize the impact of the COVID-19 pandemic as an intervention that affects society as we knew it. As the contributions in this volume will show, this intervention may further worsen the current state of affairs, accentuating its flaws and deficiencies, or possibly lead to a new and better situation. Currently, many people are concerned about the future. The introduction of lockdowns and social distancing measures makes people feeling depressed and sometimes overtly resentful of the “new normal” as it is currently being referred to. In the Netherlands, the terms “1.5-m society” and “1.5-m economy” were coined, indicating the physical requirements and limitations after the initial lockdown that might remain in place for a long time, perhaps even permanently, just like wearing a mask on certain occasions. We conceptualize the pre-COVID-19 era as the “old common” and explore the possible transition to a “new common,” with or without a coronavirus.

The word “common” has several meanings as an adjective and as a noun (Merriam Webster Dictionary 2020). It relates to a community at large or public work for the common good; to a place or common resource belonging to or shared by more individuals; to a familiar insight or widespread general knowledge as in common sense; to a piece of land subject to common use, such as a general public space like a public open area in a municipality.

In this volume, we use the word common in a variety of meanings as indicated above with a general emphasis on shared values and resources both in an abstract metaphorical sense as in a real-life physical sense. We happened to find an interesting interpretation of the phrase “new common” in a description of a community space that may be booked by the residents and organizations that work in and near St James Town in Toronto. The St. James Town website (2020) explains the term New Common as follows.

The name … was chosen to best communicate what we hope will happen in the space-people working together for the common good of everyone living in the community. This points to how The New Common is also an approach to living as a community characterized by relationship, collaboration, diversity, creativity, and empowerment.

In fact, from a sociological perspective, humans as a species always have something in common, even in a slave society occupied by masters and slaves. Therefore, when deliberating a new common, one has to give a view on the “old” common. The issue is not about having or not having a common or a society, but it is about the quality and scope of that common.

The American ecologist Garret Hardin (1968) wrote about the tragedy of the commons arguing that individuals will always try to maximize their own gains even at the cost of the common good. He already indicated a typical human feature that would later on be called short-termism, the problem of balancing the needs of both the long term and the short term. Buck Cox 1985 criticized Hardin’s tragedy of the commons for its weak historical ground and rather terms that the common usage of land had been successful for many centuries. She argues that social changes and agricultural innovation, and not the behavior of the commoners, led to the demise of the commons. So Cox’s remark can be interpreted as a strong belief in the positive attitude of individuals to contribute to the common good.

Hanging on to the Old Common might be understandable or even rational, depending on who you are, where you are from, and where you live. A lot of technological, economic, and educational progress has certainly been made in human history in the era defined as the Anthropocene, which started with the industrial revolution. Various books elaborate on this perspective, including the seminal ones by Norberg (2016) and by Rosling et al. (2018). The point all these authors make is that we tend to underestimate, by ignoring facts and figures, what has actually been achieved over the years and that on average we are much healthier, wealthier, and safer than at any point in history.

## How COVID-19 Challenges the Old Common

Obviously, COVID-19, and the resulting crisis, has revealed a number of shortcomings and cracks in the old common. We see the following major ones.

Firstly, our society lacks diversity and inclusion. Many groups are either under-represented or treated unequally or even discriminated against. This applies to women, people with a migrant background, disabled persons, and people with certain sexual orientations. A pandemic crisis is often seen as a great “equalizer” as everyone could fall ill. However, in practice, the burden of the consequences of a crisis like the COVID-19 pandemic is not equally divided and typically falls on the weaker groups as various chapters in this book will show. It operates rather selectively.

Even during the lockdown, groups of migrant workers were exposed to high risks of COVID-19 infections due to poor working conditions and the lack of options to stop working or to work from home, notwithstanding the government support to companies and workers. In the Netherlands and Germany, for example, this became painfully clear in the meat industry and slaughterhouses. Recently, after Spain had lifted large parts of the lockdown restrictions, the Ségria region near Barcelona with 200,000 inhabitants had to be closed off again due to a new outbreak in sectors with many migrant workers.

Secondly, our society appears generation biased. A sociological revolution is taking place, which already started before the corona crisis, where for the first time in history new generations do not generally have better prospects than their parents or grandparents (Putnam 2016). This applies to job security, debts, pensions, the ability to buy or rent a house, and as a consequence, the impact this all has on forming relationships and families. While the elderly were without a doubt hit hardest by COVID-19 in terms of health, morbidity, and loneliness, young people were strongly affected by the restrictions regarding going out and getting together, the lockdown of their schools and education, and the economic developments. Unemployment among young workers in temporary contracts is increasing sharply, as they are the first to be made redundant (Eurofound 2020). As a consequence, a “corona generation,” “Generation C,” or a cohort of “Coronials” might develop. During one of the crisis press conferences, the Dutch Prime Minister Mark Rutte strongly encouraged the young generation to speak up.

Thirdly, our global society is weak when it comes to international solidarity. According to UNHCR, by the end of 2018, almost 70.8 million individuals were forcibly displaced worldwide because of persecution, conflict, violence, or human rights violations, a record high. The most recent number on worldwide hunger shows an incredible number of 690 million people going to bed hungry every night (FAO 2020). Migrants are at the mercy of Western governments that act in an ambivalent, uncoordinated, and self-centered way. Often refugees become political playthings. In the coronavirus crisis, many countries and regions have insufficient means and too weak an infrastructure to be able to counteract the spread of the virus, especially among certain groups, including refugees. At the same time, Western countries cannot reach consensus on support measures and regulations and some try to buy up stocks of medical products and possible medicines and vaccines.

Finally, the old common is, to a high degree, humankind-centered, bluntly ignoring the wider ecological system of the planet of which we humans are part. Since the commercial introduction of the first versions of the steam engine that could transmit continuous power to a machine in 1712 by Thomas Newcomen, humankind has entered the industrial era. In the following man-dominated Anthropocene, much has been achieved, but much has also been destroyed, wasted, and irreversibly damaged. The notions of “externalities” and ecological footprints of human behavior and the global system we have created are of recent origin and still fairly weakly developed. This is why the old common is extremely vulnerable despite all the knowledge that has been generated. COVID-19 appears a case of zoonotic diseases that start out in animals and jump to humans under certain circumstances. Various virologists have stated that a virus restores an ecosystem. In other words, the COVID-19 crisis represents a “systemic” crisis, underpinned by a capitalist, neo-classical economic system where, in the analysis of the economist Mazzucato (2019), everything that fetches a price is of value, whereas in classical economics everything that had value used to get a price.

Many of these shortcomings are rooted in two major seemingly conflicting interests or values, which can be described as global versus local and collective versus individual issues, respectively. Indeed, Krastev (2020a) recently argued that the COVID-19 pandemic is different from earlier worldwide catastrophic events because of the level of globalization that has been reached anno 2020 and because of the unprecedented level of political control that several states such as China have imposed upon its citizens. In addition, Krastev points out that the crises amplify several paradoxes such as the looming interrelational conflicts between generations, the dilemmas states are faced with in their decisions to either stimulate the economy or contain the spread of the virus to secure people’s health, and the tendency of the national government to control its citizens versus the fundamental right of personal freedom.

Krastev (2020b) terms his findings in the following seven lessons for which he assumes a European perspective.

1. The return of “big governments”: people are inclined to rely on the government to organize a collective defense against the pandemic.
2. The increasing significance of borders: the role of the nation state becomes more important to secure national interests.
3. The growing trust in scientific expertise: people are open to trusting experts and heeding the science when their own lives are at stake.
4. The potential of using big data authoritarianism: states will use digital technology to efficiently and effectively control the movement and behavior of people to fight the crisis.
5. The message leaders have to spread: to contain the pandemic, people should drastically change their way of living, and therefore recommendations to “stay calm” and “get on with life” is the wrong message.
6. The strong impact on intergenerational dynamics as the older members of society are much more vulnerable to COVID-19 and feel threatened by millennials’ visible unwillingness to change their way of living.
7. At a certain point, governments will be forced to choose between containing the spread of the pandemic at the cost of destroying the economy or tolerating a higher human cost to save the economy.

## Towards a New Common

Can we envisage a new common, particularly in these challenging times of a pandemic and major socioeconomic crisis? What will it look like and how will we get there while preserving the best of the old common? Obviously, the new common would and should be the positive mirror image of the old common. It would have to be more inclusive, more diverse, less selective, offer more leeway for the young generations, be based on the principles of precaution, leave no one behind, and acknowledge the wider ecosystem we as humankind are inseparably part of.

One optimistic belief is that we as humans will draw lessons from this enormous shock, come to our senses, and change our ways of thinking and doing, having learned our lessons well. Many commentators are not that optimistic and allude to the previous financial crisis in the years 2008–2014, where some things were changed, but many things remained unchanged. Nevertheless, the hopes are up for the scenario that the current crisis will give a strong push to developments that were already underway, such as the efforts for an energy transition.

In general, the transition from the old to the new common can be characterized as the “Second Deep Transition,” where the industrialization is considered the “First Deep Transition.” Schot et al. (2020) put it as follows.

We need a massive redirection of our systems towards a low-carbon and circular economy, based on a better balance between local and global production, new systems of peer-to-peer consumption, a sharing economy, and the development of new type of services (and commons) to replace mass production, for example, not more automobiles, but mobility as a service.

Clearly, being able to make this transition is a matter of resilience, which should not be merely understood as the capacity to “bounce back” to the original state, but also the ability to anticipate changes and, in particular, to innovate (Wilthagen and Bongers 2020).

A recently published McKinsey report (Sneader and Singhal 2020) outlines the path to the next normal beyond the coronavirus crisis in the following five phases: resolve, resilience, return, reimagination, and reform. Defining a new common is no less than a long-term process of reimagination and reform. It is not at all a slam-dunk case. Vested interests and power relations represent strong hurdles in taking the next steps.

Various philosophical, legal, and sociological approaches have tried to pin down the ideal of a community based on good values. A case in point is “communitarianism” as promoted by authors such as Etzioni (2003) that gained attention at the turn of the millennium by stating that

Communitarianism is a social philosophy that maintains that society should articulate what is good–that such articulations are both needed and legitimate. Communitarianism is often contrasted with classical liberalism, a philosophical position that holds each individual should formulate the good on his or her own … Communitarians examine the ways shared conceptions of the good (values) are formed, transmitted, justified, and enforced.

So where should we place our bets when it comes to shaping a new common and what are the game changers? Certainly, one of the interesting solution areas can be found in the potentials of the digital transformation. More than a decade ago, Benkler (2006) asserted in his book *The Wealth of Networks* that, with the rise of the Internet and the upcoming digitalization, a new economic system based on commons becomes possible again as cheap computing power in conjunction with global communication networks will enable people to produce valuable products through non-commercial processes of interaction: “as human beings and as social beings, rather than as market actors through the price system.” Blenkler coined the term “networked *information econom*y” to refer to a “system of production, distribution, and consumption of information goods characterized by decentralized individual action carried out through widely distributed, nonmarket means that do not depend on market strategies.” He also introduced the term “*commons-based peer production*” for collaborative efforts based on sharing information. Current examples of commons-based peer productions are free and open source software platforms. We argue that the networked information economy will become the driver of the digital transformation in the new common. The ubiquitous availability of data in combination with the unlimited power of smart algorithms creates the possibility to drive the development of a new and unprecedented form of artificial intelligence, which will shape the new common.

The “Big Data Revolution” as described by Mayer-Schönberger and Cukier (2013) and Kolb (2013) embodies a promise that may help us as humans to transcend our disabilities. We have severe limitations in observing gradual and longitudinal change, rather than sudden shocks. The inroads SARS and coronaviruses have been making represent an example. In addition, our capacity to consider and understand interaction effects among a huge number of variables is low, just like our speed of calculating. Watson, the IBM supercomputer, and the game computers Deep Blue and AlphaGo have made this painfully clear. Big data and smart technologies might help us to avoid the tragedy of the commons, by showing us real time, or even ex-ante, what the collective—say common—the impact is of our individual preferences and actions, rather than the dramatic ex-post evaluations that we are making now.

We rapidly develop a digital society by virtue of all the smart devices, applications, and platforms the digital technology enables. We work from home using collaborative working environments like MS Teams, Zoom, Skype, and what have you. Smart mobile apps are rolled out with tracking and tracing functionalities. Predictive analytics are used to predict local breakouts and forecast potential scenarios. Robots are currently positioned at airfields and hospitals to check people’s temperatures. Wearable devices are introduced to alert workers when they get too close to each other. Social media are applied to replace face-to-face and physical contact with novel ways to share our emotions and feelings with our beloved ones but also with a larger, often anonymous crowd.

To put it in general terms, the coronavirus crisis is accelerating the digital transformation, at the level of individuals, at the level of our society, and even at the level of our planet. Harari (2017) convincingly argues in his most recent book Homo Deus that the powers of big data and smart algorithms are currently at work and that they will shape the twenty-first century into an all-encompassing information society.

Lovelock (2019) takes the ideas of a future information society even further, alluding to the power of the digital transformation at a global systems level. He recognizes that artificial intelligence and its supreme power and knowledge carry the potential to lead us from the current Anthropocene into the new age of the “Novacene.” For the time being, “cyborgs” will work side by side with us humans—a new and very uncommon common—but at a given moment, they will take over our tasks to best service our old planet’s ecosystem, “keeping Earth cool to fend off the heat of the sun and safe us from the worst effects of future catastrophes.” Eventually, Lovelock forecasts that the cyborg will take over the planet and leave it because life on earth is no longer possible due to the increasing heat of the evolution of the sun as a dying star.

All these ideas of a new common are compelling and frightening at the same time as the all-encompassing artificial superintelligence might not turn out to be a “blessing in device,” but could merely prove to be a “devil in device” (Wilthagen and Schoots 2019). We have to ensure that the digital transformation serves our lives as long as possible by enhancing our well-being and welfare. In his seminal book, Bostrom (2014) elaborates on the dangers of this human-made superintelligence from an ethical, legal, and societal perspective in order to stimulate the debate on a human-centric artificial intelligence. An essential precondition for a new common that will turn out better than the old common, even in a society that faces severe restrictions due to the current virus or new viruses, concerns the alignment of technology and human values, resulting in “responsible AI” (Dignum 2019).

The final question for now is how to proceed from here? There is no readily available roadmap for the new common, but we might want to use the seventeen Sustainable Development Goals (2020) defined by the United Nations in 2015 as a benchmark and guideline. The Sustainable Development Goals (SDGs) are the blueprint to achieve a better and more sustainable future for all. They address the global challenges we face, including those related to poverty, hunger, water management, inequality, climate change, environmental degradation, peace, and justice. They are all interconnected, and, in order to leave no one behind, it is important that we achieve them all by 2030. Evidently, these goals can serve the purpose of providing humankind with a meaningful pathway into the future (O’Connor 2018). The indicators connected with the SDGs should be translated into strategic program and action perspectives for all relevant societal organizations to guarantee the possibility of a significant contribution to a new common.

The SDGs can drive change and offer a narrative and an opportunity for all to speak in one language on sustainability in the broadest sense. By following the SDGs, opportunities abound for business and capital to unlock markets that offer endless potential for profit and prosperity while at the same time working towards a sustainable future. Hoek (2018) describes how this much needed “Trillion Dollar Shift” can be achieved. Vinuesa et al. (2020) discuss the critical role of human-centric artificial intelligence in achieving the Sustainable Development Goals.

Other non-exhaustive strategies for the pathway to a new common might include a further stress on the role of the region in the political, economic, and social governance of society (“glocalization”), taking the human measure and scale as the point of departure and recognizing that people are currently not well served by fragmented and non-integral systems. These systems ignore that a person performs different roles—being an inhabitant of a region, but also a worker, a parent, a patient, a consumer, et cetera—but is in essence indivisible (de Sousa Santos 2002).

For universities, there is a special role with respect to Goal 17 “Partnerships for the goals” as they can play an excellent role as drivers of regional innovation ecosystems connecting local governments, industry, citizens, and knowledge institutions in so-called quadruple or multi-helix configurations (Etzkowitz and Zhou 2013; Peris-Ortiz et al. 2016). We see this as a new primary function of so-called “fourth generation universities” in addition to the existing three primary functions education, scientific research, and impact creation.
